# SARS-CoV-2 reinfection in a healthcare worker: First case in Portugal confirmed by viral genome sequencing

**DOI:** 10.1097/j.pbj.0000000000000171

**Published:** 2022-02-08

**Authors:** Cláudio Nunes-Silva, Sara Pereira, Gabriela Canelas, Nicole Pedro, Paulo Fernandes, Luísa Pereira, Margarida Tavares

**Affiliations:** a Department of Infectious Diseases and Emerging Infectious Diseases Unit, Centro Hospitalar Universitário São João, Porto, Portugal; b FMUP – Faculdade de Medicina da Universidade do Porto, Porto, Portugal; c i3S – Instituto de Investigação e Inovação em Saúde, Universidade do Porto, Porto, Portugal; d Ipatimup – Instituto de Patologia e Imunologia Molecular, Universidade do Porto, Porto, Portugal; e ICBAS – Instituto de Ciências Biomédicas Abel Salazar, Universidade do Porto, Porto, Portugal; f CISAS – Instituto Politécnico de Viana do Castelo, Viana do Castelo, Portugal; g EPI Unit – Instituto de Saúde Pública da Universidade do Porto, Portugal.

**Keywords:** COVID-19, healthcare worker, immunity, reinfection, SARS-CoV-2

## Abstract

Few reports of SARS-CoV-2 reinfection by antigenically similar variants are well documented. The interplay between natural acquired immunity, escape by emerging variants, and protective measures in the healthcare setting is considered in this description of the first phylogenetically confirmed SARS-CoV-2 reinfection in Portugal.

## Introduction

On August 2020 the first case of re-infection by a phylogenetically distinct variant of SARS-CoV-2 was reported and, since then, an increasing number of cases have been recognized worldwide. However, well documented reports of reinfection with SARS-CoV-2 virus remain relatively uncommon.^1,2^

Studies have shown that most COVID-19 patients, regardless of the severity of infection, develop long-lasting SARS-CoV-2 specific adaptive immunity for as long as 8 months,^3^ and a prospective cohort study of antibody responses following symptomatic SARS-CoV-2 infection in healthcare workers has demonstrated that >95% of them had persistent detectable spike protein antibodies up to 200 days postinfection and at least 95% of individuals were predicted to remain seropositive to S-antibody at 465 days in a mathematical model.^4^ Reinfection was investigated in a large cohort of antibody-positive individuals who were followed for up to 35 weeks and it was shown to be a rare phenomenon with natural infection eliciting protection with an efficacy of > 90%.^5^ In another study, the SARS-CoV-2 Immunity and Reinfection Evaluation, in which data on 25,661 health-care workers in the UK (8278 of whom with known past COVID-19) was analysed, it was demonstrated that previous SARS-CoV-2 infection provided an overall 84% reduction of reinfection, reaching 93% for symptomatic infections.^6,7^ It remains unknown why these cases of reinfection by antigenically similar variants occur.

Identification of reinfection cases relies on SARS-CoV-2 molecular detection at 2 different time points and on viral genetic sequencing data to support reinfection instead of persistent viral carriage.^8^ According to the Center for Disease Control and Prevention the following criteria are used to define reinfection: 1) SARS-CoV-2 RNA detection with cycle threshold (Ct) values < 33 if detected by RT-PCR > 90 days after the first detection of viral RNA, regardless of symptom presence, and paired respiratory viral specimens from each episode belonging to 2 different clades/lineages of virus or genomes with >2 nucleotide differences per month; 2) detection of SARS-CoV-2 RNA > 45 to 89 days after first infection in a close contact of a confirmed case or in a patient with COVID-like symptoms with no alternative explanation and with laboratory evidence of reinfection (ie, Ct values and sequence genetic diversity of paired samples) as noted above.^9^

## Case report

We present the case of a previously healthy female healthcare worker in a primary care centre in Portugal. During the first COVID-19 episode, she presented with dry cough immediately followed by headache, odynophagia, anosmia, dysgeusia and myalgias, and tested positive for SARS-CoV-2 RT-PCR on a naso-oropharyngeal swab specimen on September 22, 2020. Exposure history concerned 13 persons from 4 generations of a family who gathered during a holiday week. The first identified case was her asymptomatic brother-in-law detected during a routine workplace screening, the same day of our patient symptoms’ onset. Besides these 2 cases, other 7 family members later tested positive for SARS-CoV-2, but only the patient and her mother developed symptomatic disease. Fourteen days after testing positive for COVID-19, symptoms completely subsided, she was discharged from isolation after a negative RT-PCR test and resumed work. Forty-eight days after primary infection, she again developed COVID-19-like symptoms similar to those experienced previously. Exposure history disclosed a recent close contact for >15 minutes while wearing a surgical mask during care of a COPD patient with respiratory symptoms not wearing a mask during physical examination and who was diagnosed with COVID-19. A SARS-CoV-2 RT-PCR on a naso-oropharyngeal specimen was positive on November 12. No laboratory abnormalities, including immunocompromising conditions, were found. Antibody testing after the first COVID-19 episode was not performed but anti-SARS-CoV-2 IgG antibodies were detected by a chemiluminescent microparticle assay (Abbott Diagnostics, Chicago, IL) 11 days into the second disease episode.

## Results

**Figure 1:**
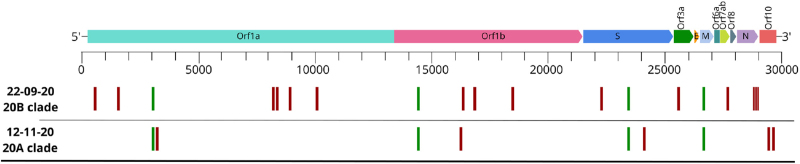
Detailed sequence diversity of the SARS-CoV-2 isolates from the 2 patient samples (date of sample collection is indicated as DD-MM-YY). On top the representation of the SARS-CoV-2 genome (genes are indicated) and bellow the nucleotide substitutions (in green shared and in red distinct substitutions between the 2 isolates) in relation to SARS-CoV-2 reference genome (NC_045512.2).

Swab samples from the 2 disease episodes were available. Multiplex RT-PCR Ct values of the 3 target gene sequences (ORF1ab, N, and E) from each disease episode specimens were 31/33/25 and 30.5/29.8/31.2, respectively. Viral whole genome-sequencing (with the Ion AmpliSeq™ SARS-CoV-2 Research Panel; Thermo Fisher Scientific, Waltham, MA), phylogenetic analysis and lineage/clade affiliation was performed as previously described.^10^ The sequencing of the viral genome of the paired samples (Fig. 1) revealed 2 different variants affiliated in 2 different clades: 20B in the first infection and 20A in the second. The 2 variants shared 4 nucleotide substitutions in relation to SARS-CoV-2 reference genome (NC_045512.2), but diverged by 20 nucleotide substitutions located throughout the SARS-CoV-2 genome. This rate of divergence exceeds largely the low rate of mutation described for SARS-CoV-2, and the fact that the viral specimens were affiliated in 2 distinct clades further supports the occurrence of reinfection.

## Discussion

Viruses, such as SARS-CoV-2, that infect mucosal surfaces and do not have a viraemic phase, result in transient antibody responses that are detected for months or a few years.^11^ Despite studies of neutralizing antibody responses against SARS-CoV-2 spike protein showing a decline in titres over time (being detectable in some studies for at least 5 months after primary infection), the long-term duration of antibody responses is not yet fully known.^12^ On one hand, failure to detect antibodies in serum in an individual after natural infection or even vaccination does not mean that they will not rapidly produce antibody upon re-exposure to the same pathogen, which is attributed to memory B cells and long-lived plasma cells, with a more rapid and stronger response and thus mitigating disease severity or preventing reinfection altogether.^12^ On the other hand, the presence of neutralizing antibodies during primary infection may be insufficient to confer protection against reinfection, as recently illustrated by a case of a solid organ transplant recipient with COVID-19, whose underlying immune deficiencies (ie, low naïve T CD4+ pools) resulted in a poor neutralizing antibody quality insufficient to protect against SARS-CoV-2 reinfection.^2^ To date it is not yet clear if SARS-CoV-2 reinfections are isolated events in individuals unable to mount an effective adaptive immune response, and ultimately what is required to develop strong humoral responses, or if they will be seen more frequently in the setting of newly emerging variants with the potential to evade immune response. Importantly, regarding this last point, in the SARS-CoV-2 Immunity and Reinfection Evaluation study, the variant of concern B.1.1.7 (renamed as Alpha variant) circulated during the observation period, causing nearly 50% of all infections, although not seeming to have an influence on the rates of reinfection.^6^

When it comes to shaping the future dynamics of SARS-CoV-2 circulation, a key aspect of reinfections pertains their infectiousness. Possible infectiousness means herd immunity from infection or vaccination is unlikely to be sufficient to eliminate the virus if reinfection cases are seen more frequently. As such, current focus should be placed not only in comparing immunity following natural infection and that elicited by vaccination to better define vaccine schedules, but also on ensuring personal protective measures in the highly exposed healthcare setting are not overlooked even after acquired immunity, by natural infection, vaccination or both, at least until more is known about the type and duration of protection they confer.

## Acknowledgements

The authors would like to thank the healthcare professional for her willingness to collaborate, critical approach and the accuracy with which she provided us with her data, and also for consent.

## Conflicts of interest

The authors have no conflicts of interest to disclose.

This case report was presented at the 9° Congresso Pandemias na Era da Globalização” in May 2021.

## References

[1] To KK Hung IF Ip JD COVID-19 re-infection by a phylogenetically distinct SARS-coronavirus-2 strain confirmed by whole genome sequencing. Clin Infect Dis. 2020;ciaa1275 doi: 10.1093/cid/ciaa1275. 10.1093/cid/ciaa1275 PMC7499500 32840608

[2] Klein J Brito A Trubin P Longitudinal immune profiling of a SARS-CoV-2 reinfection in a solid organ transplant recipient. Res Sq [Preprint]. 2021;rs.3.rs-405958doi: 10.21203/rs.3.rs-405958/v1.

[3] Dan JM Mateus J Kato Y Immunological memory to SARS-CoV-2 assessed for up to 8 months after infection. Science. 2021;371:eabf4063. 33408181 10.1126/science.abf4063 PMC7919858

[4] Grandjean L Saso A Ortiz AT Long-Term Persistence of Spike Antibody and Predictive Modeling of Antibody Dynamics Following Infection with SARS-CoV-2. Clin Infect Dis. 2021;ciab607 doi: 10.1093/cid/ciab607. 10.1093/cid/ciab607 PMC8994590 34218284

[5] Abu-Raddad LJ Chemaitelly H Coyle P SARS-CoV-2 antibody-positivity protects against reinfection for at least seven months with 95% efficacy. EClinicalMedicine. 2021;35:100861. 33937733 10.1016/j.eclinm.2021.100861 PMC8079668

[6] Hall VJ Foulkes S Charlett A SIREN Study Group. SARS-CoV-2 infection rates of antibody-positive compared with antibody-negative health-care workers in England: a large, multicentre, prospective cohort study (SIREN). Lancet. 2021;397:1459–1469. 33844963 10.1016/S0140-6736(21)00675-9 PMC8040523

[7] Krammer F. Correlates of protection from SARS-CoV-2 infection. Lancet. 2021;397:1421–1423. 33844964 10.1016/S0140-6736(21)00782-0 PMC8040540

[8] Babiker A Marvil CE Waggoner JJ Collins MH Piantadosi A. The importance and challenges of identifying SARS-CoV-2 reinfections. J Clin Microbiol. 2021;59:e02769–e2820. 33361342 10.1128/JCM.02769-20 PMC8092746

[9] Center for Disease Control and Prevention. Investigative criteria for suspected cases of SARS-CoV-2 reinfection (ICR). CDC; 2020. Available at: https://www.cdc.gov/coronavirus/2019-ncov/php/invest-criteria.html (accessed on April 18, 2021).

[10] Pedro N Silva CN Magalhães AC Dynamics of a dual SARS-CoV-2 lineage co-infection on a prolonged viral shedding COVID-19 case: insights into clinical severity and disease duration. Microorganisms. 2021;9:300. 33540596 10.3390/microorganisms9020300 PMC7912897

[11] Cohen JI Burbelo PD. Reinfection with SARS-CoV-2: implications for vaccines. Clin Infect Dis. 2020;ciaa1866 doi: 10.1093/cid/ciaa1866. 10.1093/cid/ciaa1866 PMC7799323 33338197

[12] Wajnberg A Amanat F Firpo A Robust neutralizing antibodies to SARS-CoV-2 infection persist for months. Science. 2020;370:1227–1230. 33115920 10.1126/science.abd7728 PMC7810037

